# miR-190-5p in human diseases

**DOI:** 10.1186/s12935-019-0984-x

**Published:** 2019-10-07

**Authors:** Yue Yu, Xu-Chen Cao

**Affiliations:** 1 The First Department of Breast Cancer, National Clinical Research Center for Cancer, Tianjin Medical University Cancer Institute and Hospital, Huan-Hu-Xi Road, Hexi District, Tianjin, 300060 China; 2 Key Laboratory of Cancer Prevention and Therapy, Tianjin, 300060 China; 3 Tianjin’s Clinical Research Center for Cancer, Tianjin, 300060 China; 4 Key Laboratory of Breast Cancer Prevention and Therapy, Tianjin Medical University, Ministry of Education, Tianjin, 300060 China

**Keywords:** miR-190, Cancer, Tumorigenesis, Progression, Biomarker

## Abstract

miRNAs, a major class of small noncoding RNAs approximately 18–25 nucleotides in length, function by repressing the expression of target genes through binding to complementary sequences in the 3′-UTRs of target genes. Emerging evidence has highlighted their important roles in numerous diseases, including human cancers. Recently, miR-190 has been shown to be dysregulated in various types of human cancers that participates in cancer-related biological processes, including proliferation, apoptosis, metastasis, drug resistance, by regulating associated target genes, and to predict cancer diagnosis and prognosis. In this review, we summarized the roles of miR-190-5p in human diseases, especially in human cancers. Then we classified its target genes in tumorigenesis and progression, which might provide evidence for cancer diagnosis and prognosis, promising tools for cancer treatment, or leads for further investigation.

## Introduction

Cancer is a major threat to human health worldwide and is the second leading cause of death in China [1]. Most cancer-related deaths are due to the development of metastasis, which is a multistep process where primary tumor cells disseminate from their site of origin and seed secondary tumors at a distant site [2]. However, the underlying molecular mechanisms of metastasis remain unclear. Accumulating evidence indicates that microRNAs (miRNAs) are aberrantly expressed in many types of human cancers and contribute to carcinogenesis and cancer metastasis [3]. Furthermore, miRNAs have been proposed as novel noninvasive and cost-effective biomarker screening tools, especially when applied in conjunction with ultrasound/CT/MRI, to more accurately and specifically diagnose and predict the prognosis of cancers [4, 5].

miRNAs are a major class of small noncoding RNAs approximately 18–25 nucleotides in length that execute their biological functions by repressing the expression of target genes through binding to complementary sequences, which is in the 3′ untranslated regions (UTRs) of the mRNAs of the target genes [6]. One miRNA can target multiple mRNAs; conversely, a single 3′-UTR has multiple miRNA binding sites. According to the miRbase database, over 2500 mature miRNAs have been identified in humans; however, their functions are still under investigation [7]. miRNAs play active roles and control multiple critical pathways in the regulation of most cellular processes, including proliferation, differentiation, development, apoptosis, migration, metabolism, angiogenesis, and epithelial–mesenchymal transition (EMT) [8]. The first correlation between cancer and miRNA was described as the downregulation and frequent deletions of the miRNA-encoding gene clusters of miR-15 and miR-16 in chronic lymphocytic leukemia [9]. Abnormal miRNA expression is observed in different types of cancers and has been explained by various potential mechanisms [8, 10]. Further illumination of the association between miRNAs and tumorigenesis can lead to the identification of potential diagnostic and prognostic biomarkers; thus, the identification and characterization of miRNAs and potential target genes will result in greater progress in clinical applications.

miR-190 (Gene ID: 406965) is located on the proximal end of the long arm of human genome chromosome 15 (15q22.2), which has two main mature form, including miR-190-5p and miR-190-3p. Recently, a growing body of experimental evidence suggests a clinical association between miR-190 and human diseases, notably in cancer development and progression. However, accumulating evidence indicates that miR-190-5p could play a dual role in tumorigenesis and progression. In this review, we discussed the function of miR-190-5p in human diseases, particularly in human cancers, and classified its target genes in governing cancer-related phenotypes, such as proliferation, apoptosis, metastasis, and drug resistance. We also address the clinical value of miR-190-5p as a biomarker for cancer diagnosis and prognosis and as a molecular target or promising tool for cancer therapy.

## Evidence acquisition

PubMed and Google Scholar were used to search for articles published up to September 2019 using the following keywords: miR-190, miR-190-5p, microRNA-190-5p, tumor, cancer, and carcinoma. All recognized studies were assessed for relevance by two authors by checking the title and abstract. All irrelevant articles, studies without access to the full text of the publication, letters, expert opinions, case reports, meeting proceedings, non-English articles, review articles, as well as articles whose methods do not contain biomedical experimental validation were excluded. The full text of any selected article was reviewed independently by our two authors. We also searched the reference lists of the reviewed articles to identify additional relevant articles.

## Biogenesis of miR-190

miRNAs are a large family of short noncoding RNAs that mediate posttranscriptional gene silencing by affecting both the translation and stability of mRNAs–miRNAs [8]. Similar to other miRNAs located within the intronic regions, the miR-190 encoding gene is embedded with the protein coding gene Talin2 and, utilizing the host transcription start site, is transcribed by RNA polymerase II as long primary sequences called the primary transcript (pri-miR-190) [11]. However, Chu et al. and our previous study indicated that, in breast cancer, miR-190 has its own transcriptional regulatory elements distinct from those associated with the promoter of its host gene [12, 13]. Following its transcription, pri-miR-190 is cleaved by the nuclear RNase III Drosha to produce an approximately 100 bp stem-loop precursor miR-190 (pre-miR-190), which is further exported to the cytoplasm by exportin 5 and cleaved by Dicer near the terminal loop, liberating a small RNA duplex [14]. Following Dicer processing, the RNA duplexes are loaded onto proteins from the Argonaute family to form an effector complex known as the RNA-induced silencing complex (RISC). For most miRNAs, the pre-RISC quickly removes the passenger strand (which is a known miRNA*) to generate a mature RISC. However, both strands of miR-190 can generate two mature miRNAs—miR-190-5p and miR-190-3p. A seed region at the 5′ end of miR-190-5p/3p allows either perfect or imperfect base pairing with the 3′-UTR and commonly results in translational inhibition or destabilization of the target mRNAs, respectively [8]. Little is known about the role of miR-190-3p; therefore, the current review discusses the biological function of miR-190-5p.

## miR-190-5p in cancer development and progression

**Fig. 1:**
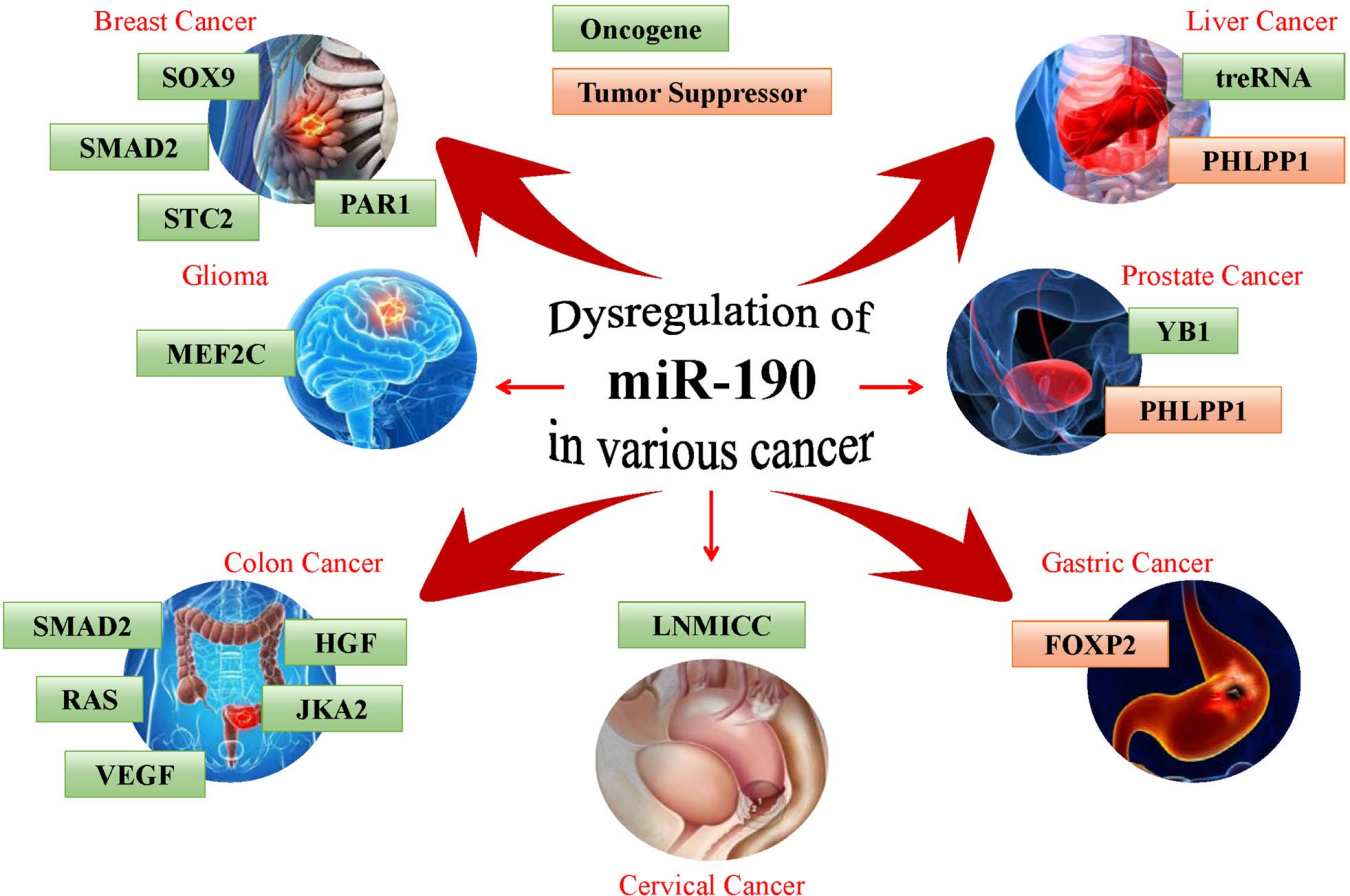
The function and validated target genes of miR-190 in human cancers

Dysregulation of miRNAs is involved in cancer initiation and progression [6]. miR-190-5p has been reported to function as both a tumor suppressor and oncogene in multiple human cancers. Upregulation of miR-190-5p was observed in pancreatic cancer [15], bladder cancer [16], meningioma [17] and gastric cancer [18], whereas downregulation of miR-190-5p was found in breast cancer [12], hepatocellular carcinoma [19], glioma [20], prostate cancer [21], rectal cancer [22] and cervical cancer [23]. These observations suggested that miR-190-5p might target multiple genes related to tumor development and progression (Table 1 and Fig. 1).

**Table 1:** Roles of miR-190 and its targets in human cancers

Cancer type	Target genes	Biological function	References
Tumor suppressor			
Breast cancer	SMAD2	Inhibiting invasion and metastasis	[12]
	SOX9	Enhancing endocrine therapy sensitivity	[24]
	STC2, PAR1	Inhibiting migration and angiogenesis	[13, 25]
Cervical cancer	–		[23]
	LNMICC	Inhibiting metastasis	[26]
Colon cancer	HGF, VEGF, JAK2, RAS, SMAD2	Inhibiting angiogenesis	[27]
Glioma	MEF2C	Inhibiting proliferation, migration, and invasion and promoting apoptosis	[20]
	–	Inhibiting tumor growth	[28]
Hepatocellular carcinoma	treRNA	Inhibiting migration and invasion	[19]
Prostate cancer	YB1	Inhibiting proliferation	[21]
Rectal cancer	–		[22]
Oncogene			
Gastric cancer	FOXP2	Promoting proliferation, migration and invasion	[18]
Prostate cancer	PHLPP1	Promoting migration and invasion	[29]
Hepatocellular carcinoma	PHLPP1	Promoting proliferation, migration and invasion	[30]

### miR-190-5p in proliferation

Uncontrolled cell proliferation is one of the hallmarks of cancer [31]. Increasing evidence indicates that miR-190-5p associates with several genes involved in cancer proliferation. Y-box binding protein-1 (YB-1), an oncogenic transcription or translation factor, was identified as a marker of malignant cell transformation and tumor aggressiveness and as a potential molecular target for cancer therapy [32]. The functions of YB-1 in prostate cancer are related to cell proliferation [33]. Xu et al. [21] disclosed that miR-190-5p inhibited prostate cancer proliferation by targeting YB-1. In addition, miR-190-5p was also demonstrated to be downregulated in glioma, and overexpression of miR-190-5p inhibited glioma cell growth by targeting myocyte enhancer factor 2C (MEF2C) [20, 28], which functions as a transcription factor of muscle-specific genes during skeletal muscle terminal differentiation [34]. Several studies have revealed an oncogenic role for MEF2C in different types of human cancers [35, 36]. miR-190-5p inhibits cell proliferation in glioma by inhibiting the MEF2C-JAGGED1-Notch signaling axis [20].

Conversely, miR-190-5p exerts an opposing role in hepatocellular carcinoma (HCC) and gastric cancer [18, 30]. Cell proliferation was enhanced in HepG2, Hep3B and BEAS-2B cells with stable miR-190-5p overexpression compared to control cells [30, 37]. AKT is a serine/threonine protein kinase that plays critical roles during tumorigenesis and progression [38]. PH domain and Leucine Rich Repeat Protein Phosphatase 1 (PHLPP1), a member of the PHLPP family, directly dephosphorylates and therefore inactivates AKT to inhibit cancer proliferation [39]. PHLPP1 was identified as a target of miR-190-5p, and miR-190-5p overexpression led to higher levels of pAKT, which was correlated with PHLPP1 as evidenced by the fact that elevating PHLPP1 expression resulted in reduced pAKT levels [30, 37]. Another study demonstrated that miR-190-5p was increased in gastric cancer and functioned as an oncogene, which is based on the contribution of miR-190-5p to the proliferation of SGC7901 cells via targeting FOXP2 [18]. Interestingly, benzo[a]pyrene promotes the proliferation of normal cells and exacerbates apoptosis signaling in cancer cells. BP-induced upregulation of miR-190-5p was detected in normal cells accompanied with downregulation of mRNA levels of TP53INP1 and PHLPP1 genes, which demonstrates that miR‐190-5p is possibly involved in the general cellular response to benzo[a]pyrene [40]. These findings show that the relationship between miR-190-5p and cell proliferation is complex and that miR-190-5p might have different effects in various types of human cancers, which requires further investigation to address these discrepancies.

### miR-190-5p in apoptosis

Accumulating evidence indicates that dysregulation of apoptosis is related to most diseases, all of which involve multiple signaling transduction pathways [41]. p53 is a transcription factor responsible for the transcriptional regulation of genes involved in cell cycle progression, DNA repair and apoptosis [42]. NFκB1/p50 modulates p53 expression by regulating the miR-190-PHLPP1-AKT/S6 ribosomal protein pathway, and the introduction of miR-190-5p into p50−/− cells restored the cells’ apoptotic response following arsenite exposure [43]. Furthermore, miR-190-5p promotes apoptosis in the glioma cell lines U251 and U87 by targeting MEF2C [20].

### miR-190-5p in metastasis

Metastasis is the leading cause of cancer-related mortality among patients with cancer. Cancer metastasis begins with detachment of metastatic cells from the primary tumor followed by the travel of the cells to different sites through the circulatory/lymphatic systems and finally settlement and growth of the cells at a distal site [44]. During the process, metastatic cells undergo detachment, migration, invasion and adhesion. These four essential metastatic steps are interrelated and affected by multiple biochemical events and parameters [45]. EMT has been shown to play pivotal roles in these steps to promote metastasis. Transforming growth factor-β, (TGF-β), a key driver of EMT, plays an important role in cancer metastasis [46]. In TGF-β-activated cells, SMAD2 and SMAD3 form complexes with SMAD4 and then translocate into the nucleus to regulate the expression of target genes [47]. Our previous study indicated that SMAD2 is a target of miR-190-5p and that miR-190-5p suppresses breast cancer metastasis by regulating TGF-β-induced EMT [12]. Consistent with this, other studies also indicated that miR-190-5p suppresses cell migration, invasion and an EMT-like phenotype by targeting STC2 or PAR1 in breast cancer [13, 25]. Moreover, miR-190-5p inhibits TGF-β signaling in the lung adenocarcinoma cell line A549 [48]. Vascular endothelial growth factor is a major contributor to angiogenesis, a vital process in tumor metastasis. miR-190-5p significantly suppresses tumor metastasis and angiogenesis by governing a large group of angiogenic effectors, including TCF4, SMAD2, SMAD4, RAS2, JAK2, IGF1, and HGF [27]. Consistent with our observation in breast cancer, miR-190-5p overexpression inhibits cell migration and invasion and reverses TGF-β-induced EMT in the HCC cell lines HepG2 and Huh7 by targeting the long noncoding RNA treRNA [19]. However, the contrary point of view has been reported by other scholars, whose result showed a promoting metastasis function of miR-190 in HepG2 cell lines [30]. Since the lack of some relevant research works, the issue remains controversial, which needs further researches. Despite this, it’s still undeniable that miR-190-5p functions as a promising antitumor target for clinical applications.

Clusterin, a small heat-shock-like protein, is overexpressed in many solid tumors and regulates the PI3K/AKT pathway. Clusterin was shown to dramatically enhance the migratory and invasive behavior of the normal prostate epithelial cell line PNT1A and the prostate cancer cell line PC3 by regulating the miR-190-5p-PHLPP1 axis, suggesting that miR-190-5p functions as an oncogene in prostate cancer [29]. Furthermore, miR-190-5p promotes cell migration and invasion by targeting FOXP2 in gastric cancer [18], suggesting a differential function of miR-190-5p possibly related to the specific disease context.

### miR-190-5p in drug resistance

Approximately 75% of breast cancers are hormone receptor-positive and express estrogen receptor-α (ERα) or/and progesterone receptor [49]. Therapies targeting ERα have been successfully applied in patients with ERα+ breast cancer. However, intrinsic or acquired resistance to anti-estrogen therapy presents a major challenge [50]. Sry-related high motility group box 9 (SOX9) plays active roles during tumorigenesis and progression in various types of human cancers and was reported to be upregulated in tamoxifen-resistant breast cancer and to drive breast cancer endocrine resistance [51, 52]. In our previous study, we observed that miR-190-5p increased the anti-estrogen sensitivity of breast cancer cells and identified SOX9 as a direct target of miR-190-5p [24]. Although our study indicated that miR-190-5p dysfunction contributes to endocrine therapy resistance, the function of miR-190-5p in cancer chemosensitivity still needs to be clarified in the future.

### miR-190-5p in cancer diagnosis and prognosis

Cancers often progress to middle or advanced stages at the time of diagnosis; therefore, early tumor diagnosis or screening is critical for successful treatment and improved prognosis [53]. miRNAs are strong candidates for predictive therapeutic biomarkers and early cancer biomarkers because they are stable, are easily detected and can be acquired in a minimally invasive manner [5]. Thus, miRNAs are thought to be a class of novel markers that will be commonly used to detect diseases in the coming decades. miR-190-5p expression was decreased in cervical cancer tissues in human papillomavirus (HPV)-positive patients, suggesting that miR-190-5p may be a novel diagnostic biomarker for the development of cervical cancer in high-risk HPV-positive patients [23]. High expression of miR-190-5p and low expression of miR-29c-3p and miR-219-5p are associated with notably higher recurrence rates in meningioma patients. In addition, the expression level of miR-190-5p is a prognostic predictor of postsurgical meningioma patients [17]. miR-190-5p was also downregulated in pancreatic cancer tissues compared to normal rectal mucosa [22]. Consistent with the results in other cancers, decreased miR-190-5p expression was also observed in breast cancer compared to normal breast tissue [12]. Triple-negative breast cancer (TNBC) is an aggressive subtype of breast cancer with poor prognosis. The expression of miR-190-5p was significantly lower in TNBC tissues than in normal breast tissues [54]; however, miR-190-5p expression was significantly increased in pancreatic cancer tissues and cell lines [15]. These preliminary results suggest that miR-190-5p might be an emerging biomarker of cancer diagnosis, but more clinical investigations with larger cohorts are needed.

As the mechanisms relating to the interactions between miRNAs and cancer have been gradually disclosed, miRNA could serve as a prognostic tool in cancer by estimating the patient’s overall survival, anticipating disease outcomes and predicting recurrence [8]. Several studies have indicated that miR-190-5p is a potential prognostic predictor in patients with breast cancer. As miRNAs regulate tumor progression and metastasis, dormant tumors could be distinguished from faster-growing tumors by the differential expression of miRNAs. Papadaki et al. [55] indicated that patients with early breast cancer who relapsed had lower miR-190-5p expression levels than did non-relapsed patients, and, accordingly, we showed that patients with early disease and high miR-190-5p expression in breast cancer tissues had significantly better disease-free survival and overall survival than did patients with low expression in breast cancer tissues [12]. Furthermore, higher miR-190-5p expression levels in core biopsies sampled from patients with TNBC may be associated with a better pathologic response to chemotherapy [56]. These investigations implied that miR-190-5p downregulation could serve as a biomarker for poor prognosis in patients with breast cancer.

## miR-190-5p in other diseases

In addition to the important role of miR-190-5p in human cancers, miR-190-5p plays an important role in other disease, including drug addiction, pulmonary arterial hypertension and diabetes mellitus (Table 2).

**Table 2:** Roles of miR-190 and its targets in other diseases

Disease	Target genes	Biological function	References
Drug addiction	Neuro D	Promoting drug addiction	[57]
Pulmonary arterial hypertension (PAH)	KLF15, ROCK1	Promoting pulmonary arterial hypertension	[58, 59]
Diabetes Mellitus	KRAS, SLC17A6	Inhibiting insulin resistance	[60, 61]

### miR-190-5p in drug addiction

Addiction is highly related to changes in neuronal activity, with alcohol, nicotine, caffeine, and opioids as the classic addictive drugs. The history of opioid addiction can be traced back to the first use of morphine [62]. Neurogenic differentiation 1 (NeuroD) is critical for the development of both the central nervous and endocrine systems. miR-190-5p plays an important role in the central nervous system, as indicated partly by its ability to inhibit NeuroD expression [57]. The cellular level of NeuroD is modulated differently by opioid receptor agonists [63, 64]: fentanyl increases NeuroD levels by reducing the amount of miR-190-5p, whereas morphine does not alter NeuroD levels. The different mechanisms utilized by the two agonists in activating extracellular regulated protein kinase (ERK) account for their different abilities to control miR-190-5p expression [11]. Fentanyl decreases the level of miR-190-5p, which depends on β-arrestin-mediated ERK phosphorylation and nuclear translocation of phosphorylated ERK, whereas morphine uses the PKC pathway for ERK phosphorylation and retains phosphorylated ERK in the cytosol, which renders it unable to regulate miR-190-5p levels [57]. However, miR-190-5p may have other targets in addition to NeuroD. Hence, the functions of miR-190-5p may not be limited in regulating NeuroD expression [65]. The role of miRNAs in drug addiction may open the door to possible miRNA-mediated interventions relating to opioid addiction and may be valuable targets for more efficient therapies.

### miR-190-5p in pulmonary arterial hypertension

Pulmonary arterial hypertension (PAH) is a devastating, life-threatening condition characterized by vasoconstriction and vascular remodeling. Recent studies demonstrate that miRNAs have been increasingly found in the systemic circulation of both animals and humans and suggested as great potential biomarkers for the diagnosis of various diseases [66, 67]. Hypoxia-induced changes in plasma miRNA levels correlate with PAH. miR-190-5p, which is induced in hypoxia, is necessary for Sima-dependent gene expression and promotes terminal tracheal cell sprouting [59]. miR-190-5p has been reported to affect vascular tone and calcium influx into smooth muscle cells by targeting the Kþ-channel Potassium Voltage-Gated Channel Subfamily Q Member 5 [68]. The upregulation of miR-190-5p leads to membrane depolarization (via a decrease in Kv7.5), causing calcium influx and resulting in profound vasoconstriction, which is one of the pathophysiological features of PAH [66]. Additionally, miR-190-5p is a novel regulator of the hypoxia response that represses the oxygen sensor Fatiga, leading to HIFα stabilization and enhanced hypoxic responses [69]. Specifically, miR-190-5pa-5p expression is dynamically altered in response to hypoxia and regulates hypoxia-induced PH by targeting KLF15 [58]. In addition, other scholars have reported Rho Associated Coiled-Coil Containing Protein Kinase 1 (ROCK1) as a possible target of miR-190-5p. The Rho/ROCK pathway, especially Rho-kinase isoform 2 (ROCK2), is involved in cell cycle progression, resulting in increased proliferation of pulmonary artery endothelial cells and pulmonary artery smooth muscle cells in patients with PAH [59]. However, the circulating levels of miR-190-5p correlate with the severity of PAH, and whether these levels are useful as a diagnostic or prognostic marker in PAH remains unknown.

### miR-190-5p in diabetes mellitus

Diabetes mellitus is a long‐term metabolic disease characterized by high blood glucose and insulin resistance and is frequently accompanied by cardiovascular diseases, renal failure, and visual damage [70]. miR-190-5p downregulation was observed in methylglyoxal-induced endothelial insulin resistance, which was due to increased KRAS [60]. miR-190-5p was also identified as a biomarker of insulin resistance in obese preschoolers [71]. These results suggest that miR-190-5p plays an important role in diabetes mellitus. Diabetic neuropathy is one of the most common complications of diabetes mellitus. More evidence shows that miRNAs are dysregulated and play important roles in the progression of diabetic neuropathic pain (DNP) [72]. miR-190-5p was validated to be the most significantly downregulated miRNA in DNP [73]. Moreover, miR-190-5p expression was decreased in the spinal tissue of individuals who developed DNP, and SLC17A6 is a direct target of miR-190-5p [61].

## Conclusion and future directions

In this review, we summarize the dysregulation of miR-190-5p in a variety of human diseases, especially in human cancers, highlighting the role of miR-190-5p in cancer cell proliferation, apoptosis, metastasis, and drug resistance. To the best of our knowledge, this is the first review that focuses on the role of miR-190-5p in tumorigenesis and progression as well as in clinical applications. We uncovered some interesting evidence that might be beneficial for clinical applications and future studies. Dysfunction of miR-190-5p was observed in various types of human cancers, whereas it may present different roles in identical cancers. For example, miR-190-5p functioned as a tumor suppressor in one study about HCC [19], whereas it functioned as an oncogene in another study about HCC [30]. Similarly, miR-190-5p suppresses prostate cancer proliferation [21], while it promotes cell migration and invasion in another study on prostate cancer [29]. If each study is scientific and convincing, we assume that the differences might be associated with different cancer cell lines used or experimental methods employed. Moreover, miR-190-5p does not only function as a tumor suppressor but also acts as an oncogene. It has been suggested that various miRNAs could produce tumor suppressive or oncogenic effects as a result of the suppression of both tumor suppressive and oncogenic mRNAs, and it is the balance between the multiple processes during carcinogenesis and tumor progression that ultimately determines the net function of a specific miRNA [74]. More convincing and large-scale investigations are required to confirm the detailed roles of miR-190-5p. Furthermore, we disclose that miR-190-5p has a diverse range of target genes when it functions in cancer, suggesting that the molecular mechanisms of miRNAs are extremely complicated and variable and that other targets of miR-190-5p relating to cancer need to be investigated in future. We sincerely hope that this review might provide a basis for future clinical applications and investigations.

## Data Availability

Not applicable.

## Abbreviations

DNP: diabetic neuropathic pain
EMT: epithelial–mesenchymal transition
ERK: extracellular regulated protein kinase
ERα: estrogen receptor-α
HCC: hepatocellular carcinoma
HPV: human papillomavirus
MEF2C: myocyte enhancer factor 2C
microRNA: miRNA
NeuroD: Neurogenic differentiation 1
PAH: pulmonary arterial hypertension
PHLPP1: PH domain and Leucine Rich Repeat Protein Phosphatase 1
RISC: RNA-induced silencing complex
ROCK1/2: Rho Associated Coiled-Coil Containing Protein Kinase 1/2
SOX9: sry-related high motility group box 9
TGF-β: transforming growth factor-β
TNBC: triple-negative breast cancer
UTR: untranslated region
YB-1: Y-box binding protein-1
